# Brazilian guidelines for chronic kidney disease-mineral and bone metabolism disorders in children and adolescents

**DOI:** 10.1590/2175-8239-JBN-2021-S114

**Published:** 2021-12-03

**Authors:** Ana Lúcia Cardoso Santos Abreu, Emília Maria Dantas Soeiro, Leonardo Gonçalves Bedram, Maria Cristina de Andrade, Renata Lopes

**Affiliations:** 1 Universidade Federal de São Paulo, São Paulo, SP, Brazil.; 2 Universidade Federal de Pernambuco, Recife, PE, Brazil.; 3 Instituto de Medicina Integral Professor Fernando Figueira - IMIP, Recife, PE, Brazil.

## 1. Diagnosis of chronic kidney disease-mineral and bone disorders (CKD-MBD)

1.1 Clinical Assessment 

1.1.1 In CKD children, take clinical history and perform physical examination searching for changes in CKD-MBD (Opinion).

1.1.2 The frequency of evaluation depends on the patient's age, alterations found, and CKD stage (Opinion).

## Rational

Considering the high prevalence of bone deformities, short stature and fracture in CKD children, which are not always reported, it is important to proactively inquire the patients or their families for such alterations during the anamnesis and look for them on physical examination^1^. 

## 1.2 Assessment of biochemical changes

1.2.1 We recommend assessment of serum levels of calcium (Ca), phosphorus (P), total alkaline phosphatase (AP), intact parathyroid hormone (PTH), bicarbonate (HCO3) and 25(OH)vitamin D in all children and adolescents with CKD G2-5D (Evidence).

1.2.2 The frequency of monitoring should be based on the presence and magnitude of biochemical changes, on the rate of CKD progression, according to the treatment of CKD-MBD, with the use of growth hormone or kidney transplantation (Opinion).

1.2.3 For patients with CKD G2-5D, we recommend that therapeutic decisions be based on trends, and not solely on a single laboratory value (Evidence).

1.2.4 In patients with CKD G2-5D, Ca and P levels should be kept within normal ranges for age (Evidence).

1.2.5 In patients with CKD G5D, we recommend maintaining PTH levels in the range of 3 to 5 times the upper limit of normality (Opinion).

1.2.6 In patients with CKD G2-5D, the serum HCO_3_^-^ level should be maintained between 22 and 26 mEq/L (Opinion).

1.2.7 In patients with CKD G2-5D, 25(OH)vitamin D levels should be maintained above 30 ng/mL (Opinion). 

## Rational

During childhood and adolescence there is a significant increase in bone mass, around 80%, being greater until the age of 3 and decreasing until the onset of puberty. Then it increases again and the individual reaches the peak of bone mass between 21 and 25 years old^2^ ^,^ ^3^. The progressive increase in bone mass is caused by the anabolic state characteristic of this age group. However, chronic diseases such as CKD might compromise this pattern by reducing the final bone mass. In the pediatric population, changes in bone metabolism may occur early, in CKD stage 2 already, such as bone pain and deformities, fractures and growth deficits^4^ ^,^ ^5^. Therefore, it is important to assess the bone metabolism of these children and adolescents by means of serum levels of Ca, P, AP, PTH and 25(OH)vitamin D. It is important to note that serum Ca and P values vary according to age (Table 1)^5^. Several factors, including age and sex, influence the serum AP level, which increases with bone growth and puberty. It also differs among some commercially available laboratory tests (Table 2). The AP expresses osteoblastic activity and, despite the bone fraction being more reliable, due to its high cost, we use total alkaline phosphatase^6^ ^-^ ^9^. 

**Table 1.** Normal ranges of ionized Ca, total Ca and phosphorus according to age

Age Group	Ionized Ca (mmol/L)	Total Ca (mg/dL)	P (mg/dL)
0-5 months	1.22 - 1.40	8.7 - 11.3	5.2 - 8.4
6-12 months	1.20 - 1.40	8.7 - 11.0	5.0 - 7.8
1-5 years	1.22 - 1.32	9.4 - 10.8	4.5 - 6.5
6-12 years	1.15 - 1.32	9.4 - 10.3	3.6 - 5.8
13-20 years	1.12 - 1.30	8.8 - 10.2	2.3 - 4.5

Source: Adapted from reference 5.

**Table 2.** Total alkaline phosphatase reference values according to age, sex and methodology

Age Group	Alkaline phosphatase							
	Roche Cobas		Siemens Vista		Beckman Coulter		Ortho Vitros	
	F	M	F	M	F	M	F	M
0-14 days	83 - 248	83 - 248	81.5 - 248.7	81.5 - 248.7	77 - 237	77 - 237	91 - 256	91 - 256
15 days-1 year	122 - 469	122 - 469	121.7 - 472.6	121.7 - 472.6	116 - 450	116 - 450	131 - 476	131 - 476
1-10 years	142 - 335	142 - 335	141.8 - 336.4	141.8 - 336.4	135 - 320	135 - 320	151 - 342	151 - 342
10-13 years	129 - 417	129 - 417	128.1 - 419.6	128.1 - 419.6	122 - 400	122 - 400	137 - 424	137 - 424
13-15 years	57 - 254	116 - 468	55.5 - 255.2	114.9 - 471.3	52 - 243	109 - 449	66 - 263	124 - 474
15-17 years	50 - 117	82 - 331	48.7 - 116.3	80.9 - 333.2	46 - 110	77 -317	59 - 126	91 - 339
17-19 years	45 - 87	55 -149	43.1 - 86.1	53.2 - 149.1	41 - 82	50 - 142	54 - 96	64 - 158

Source: Reference 6.

*laboratory test

** F = female; M = male

Serum PTH levels may be elevated from stage 2, progressively increasing as kidney function deteriorates, in order to maintain Ca and P levels in an appropriate range^10^ ^,^ ^11^. Optimal PTH values in the CKD patient, both for adults and children, remain a challenge, mainly due to the variability of results obtained by different PTH assays. 

The 2017 KDIGO suggests PTH levels between 2 to 9 times the upper limit of normal for children on dialysis, which corresponds to a target range of 120-540 pg/mL^11^ ^,^ ^12^. On the other hand, the European Paediatric Dialysis Working Group has shown that high-turnover disease might occur at lower PTH levels than the current guidelines suggest^13^. They reiterate the 2006 recommendations and suggest keeping PTH levels up to 2-3 fold the upper limit of normal in children on dialysis (120-180 pg/mL). Currently, some authors suggest for children with CKD stages 2-3, PTH levels 1 to 2 fold the upper limit of normal, and for children in stages 4-5D, values 1.7 to 5 fold the limit of normal. They consider that the increase in PTH would be an adaptive response to decreased kidney function in these patients, avoiding hyperphosphatemia, hypocalcemia, and calcitriol deficiency^14^. Recently, a study performed in Brazil with bone biopsies from 42 children and adolescents undergoing dialysis has shown that PTH levels lower than 2 times the normal value for age were associated with low bone turnover^15^.

In a prospective analysis, the Chronic Kidney Disease in Children (CKiD) study has demonstrated that the prevalence of metabolic acidosis positively correlated with CKD stages 2-5D^16^. The patients' serum bicarbonate levels < 18 mEq/L compared to > 22 mEq/L are associated with greater CKD progression, short stature, and increased mortality^17^ ^,^ ^18^. On the other hand, serum bicarbonate levels > 32 mEq/L also increase mortality^17^, so caution is recommended regarding rapid or excessive alkalinization, which may predispose to hypokalemia, QT interval prolongation, and cardiac arrhythmia^19^. Thus, we suggest maintaining serum bicarbonate levels between 22 and 26 mEq/L.

The term vitamin D encompasses both ergocalciferol (vitamin D2) and cholecalciferol (vitamin D3). Both undergo hydroxylation in the liver, resulting in 25-hydroxyvitamin D (ergocalcidiol and calcidiol), and subsequently a second hydroxylation occurs in the kidneys, resulting in 1,25 dihydroxyvitamin D (calcitriol), which is its active form^20^ ^,^ ^21^. CKD patients usually have hypovitaminosis D due to reduced physical activity and exposure to sunlight, decreased intake of vitamin D-rich foods, loss of vitamin D-binding protein via urine or peritoneal dialysis, among other causes^22^ ^,^ ^13^. 

Serum levels are classified as follows: sufficiency: > 30 ng/mL, insufficiency: 20-30 ng/mL, deficiency: 5-20 ng/mL, severe deficiency: < 5 ng/mL^13^. Values higher than 150 ng/mL are considered intoxication levels.

In children with CKD, there are few studies assessing the effects of 25(OH)vitamin D on bone and there is no established optimal level. However, children with 25(OH)vitamin D above 30 ng/mL have been shown to experience delayed progression of SHPT^23^, and other authors have observed that lower levels of calcium and 25(OH)vitamin D were independently associated with a reduced tibial cortical volume^24^ ^,^ ^25^. Therefore, we suggest keeping 25(OH)vitamin D levels higher than 30 ng/mL.

Regarding the periodicity of biochemical evaluation for CKD-MBD, we suggest that it should be performed according to CKD stages, from stage 2 onwards, according to Table 3 ^26^ ^,^ ^27^.

**Table 3.** Biochemistry monitoring interval according to CKD stages

CKD Stages	Calcium, Phosphorus and HCO_3_^*^	AP	PTH	25(OH)vitamin D
2 to 3	6 to 12 months	According to baseline	According to baseline	According to baseline value and therapeutic intervention
4	3 to 6 months	6 to 12 months	6 to 12 months	
5 and 5D	1 to 3 months	3 to 6 months	3 to 6 months	

Source: References 26 and 27.

## 1.3 Assessment of bone changes in CKD-MBD

1.3.1 In patients with CKD G2-5D, osteometabolic changes may be assessed by bone radiography (Opinion).

1.3.2 In patients with CKD G2-5D, it is recommended to consider bone biopsy if clinical and biochemical findings are in disagreement with each other and/or in the presence of bone deformity or pain, fragility fracture, hypercalcemia and persistent hypophosphatemia (Opinion).

## Rational

Evidence to recommend radiological assessment of bone disease in pediatric CKD is scarce. According to the International Society for Clinical Densitometry (ISCD), bone densitometry (DXA) is the method of choice for assessing bone mineral density in adults^28^. With regard to children, DXA results are not predictors of fracture risk; therefore, KDIGO 2017 does not recommend the use of this test at this age group^11^. Denburg et al. have observed a correlation between changes in tibial cortical mineral density and fracture risk^24^. Recently, a study by the *Universidade de São Paulo* group has shown an association of low bone mineral density and mineralization defects in children with CKD, assessed by DXA^15^. More recently, another study, which has assessed low bone mineral density by high-resolution peripheral quantitative computed tomography (HR-pQCT), observed no correlation with DXA findings^29^. HR-pQCT is a three-dimensional (3D) technique that measures volumetric bone mineral density and cortical bone dimensions, and analyzes the trabecular bone microarchitecture, enabling measurement of number, thickness and separation of bone trabeculae^30^. However, in 2011, Bacchetta et al. found no differences in bone parameters in 22 CKD children and adolescents, when compared to the control group of healthy children^31^. Since it is a high-cost tool, the use of HR-pQCT remains restricted to the field of scientific research. Therefore, we do not recommend routine radiological assessment for these children and adolescents. 

Recently, the European Society for Paediatric Nephrology suggests performing conventional radiography in children with bone pain, suspected nontraumatic fractures, genetic diseases with specific bone involvement, suspected avascular necrosis and proximal femoral epiphysis, and extraskeletal calcifications. In addition, they suggest radiography of the left wrist to assess bone age or knee radiography to assess the metaphyseal region in infants. On the one hand, they consider the low cost of the exam and the availability of services, and on the other hand, the low sensitivity of the exam, which depends on the experience of the radiologist for its interpretation, besides the exposure to radiation^1^.

Children and adolescents with CKD may have changes in bone turnover and mineralization^32^. Such impairments result in increased risk of fractures, pain, bone deformities, growth deficits, and affect the quality of life of these patients. Thus, the diagnosis of renal osteodystrophy is of paramount importance and, sometimes, the help of bone biopsy is needed to relate clinical and histological findings, guiding the appropriate treatment^4^. Histomorphometric analysis results in the diagnosis of renal osteodystrophy, analyzed by the TMV system (turnover, mineralization and volume)^33^. Studies in Brazilian children undergoing dialysis have shown low turnover disease in 60% of patients^15^ ^,^ ^34^, followed by a mineralization defect in one third of these children. These findings are similar to those of North American children^35^ and differ from most other studies that have shown high turnover disease^36^ ^,^ ^37^, even in early stages of CKD^36^. It is possible that the findings of low turnover result from therapies with vitamin D and its analogues. With regard to mineralization, changes are present in the early stages of CKD, and persist in dialysis patients^15^ ^,^ ^36^. These changes may remain despite treatment with calcitriol^38^.

Given the invasive nature of bone biopsy, efforts have been made in an attempt to use circulating biomarkers and imaging exams that may reflect bone turnover and mineralization. In clinical practice, high serum PTH and AP might characterize high turnover disease, while low PTH and AP suggest adynamic bone disease. However, there is no cut-off point for PTH and AP that could predict bone turnover and mineralization in these children^34^ ^,^ ^39^. Furthermore, in early stages of CKD, mineralization defects may occur even before biochemical changes^40^. Therefore, it is sometimes necessary to indicate a bone biopsy to guide the treatment.

## 1.4 Assessment of vascular calcification in CKD-MBD

1.4.1 For patients with CKD G3-5D, cardiovascular assessment by echocardiogram is recommended, and its frequency of monitoring should be according to the changes found (Opinion).

1.4.2 Echocardiogram may be used to assess the presence or absence of valve calcification, as an alternative to computed tomography (Evidence). This is recommended by KDIGO 2009 for adult patients, but is not well established in the pediatric age group. 

## Rational

Cardiovascular disease is the most important cause of morbidity and mortality for pediatric patients with CKD, with mortality up to 30 times higher than in healthy children^41^ ^,^ ^42^ ^,^ ^43^. The presence of vascular, valve and soft tissue calcification increases the risk of mortality^4^, and its prevalence increases with CKD progression.

The independent predictors of coronary artery calcification (CAC) are: dialysis vintage, elevated Ca, P, and PTH levels^44^ ^,^ ^45^ ^,^ ^46^. Another factor associated with vascular calcification is the decreased serum levels of calcification inhibitors (fetuin A and osteoprotegerin), caused by dialysis^47^ ^,^ ^48^ and the treatment of metabolic changes with Ca-based P binders and vitamin D analogues^49^, leading to hypercalcemia. 

Echocardiogram is the gold standard for accessing heart valve morphology and function, and the presence of valve calcification is a good predictor of CAC^4^_._ On the other hand, electron-beam computed tomography (EBCT) and multislice computed tomography (MSCT) have occasionally been used to assess and quantify vascular calcification^50^. Although literature proposes several methods for calcification assessment, we know that these exams are not routinely performed in pediatric clinical practice.

## 2. Treatment of CKD-MBD

2.1 Control of serum levels of Ca, P and vitamin D

2.1.1 For patients with CKD G2-5D, we suggest a 24-hour dietary recall to identify the major dietary sources of Ca and P, including P-containing additives present in processed foods (Opinion).

2.1.2 We suggest that Ca total intake (including diet, drugs, and P binders) should be within the suggested dietary intake (SDI) (Opinion).

2.1.3 For young infants or under special situations, such as persistent hypocalcemia, Ca intake may be maintained twice above the SDI, in addition to Ca supplementation, vitamin D, and/or use of high Ca dialysate (Opinion).

2.1.4 When serum Ca level is higher than the upper limit for age, it is recommended discontinuing the use of vitamin D, calcitriol, or vitamin D analogues, and replacing the Ca-based phosphate binders with sevelamer (Opinion).

2.1.5 We suggest dietary intake of P should be within the SDI for age, provided it does not compromise adequate nutrition (Opinion). 

2.1.6 For patients with CKD G2-5D and hyperphosphatemia, we suggest reducing dietary P intake to the lower limit of the SDI, without compromising nutrition (Opinion).

2.1.7 For patients with hyperphosphatemia, despite dietary P restriction, the introduction of phosphate binder is recommended (Opinion).

2.1.8 Prefer the use of calcium-based binders in the absence of hypercalcemia (Opinion).

2.1.9 For dialysis patients and those with persistent hyperphosphatemia, increasing the frequency and/or time of dialysis is recommended (Evidence).

2.1.10 For patients with persistent hypophosphatemia, it is recommended increasing dietary P intake and, if necessary, supplementing P, particularly in those undergoing daily dialysis or with renal P loss (Opinion).

2.1.11 For children with 25(OH)vitamin D deficiency or insufficiency, it is recommended correcting the changes as for the general population.

## Rational

The treatment of CKD-MBD in the pediatric population is especially difficult due to the demand on the growing skeleton. Rickets, fractures, growth deficits, secondary hyperparathyroidism, in addition to adynamic bone disease and vascular calcification^51^, may occur without adequate control of metabolic changes, increasing mortality and worsening quality of life.

According to epidemiological data, elevated phosphorus levels, and even levels within normal range, are associated with an increased risk of cardiovascular events and/or mortality^4^. The introduction of the low-P diet should be cautious, since in the pediatric population, strict dietary restrictions might lead to low bone mineralization, impairing growth^32^ ^,^ ^52^.

Recently, the Pediatric Renal Nutrition Taskforce adopted an approach of recommending Ca and P values based on the average of two standard deviations of previously published international values, referred to as the Suggested Dietary Intake (SDI)^53^ (Table 4).

**Table 4.** Suggested dietary intake for calcium and phosphorus in children with CKD 2-5D

Age	Calcium SDI (mg)	Phosphorus SDI (mg)
0 to 4 months	220	120
4 to 12 months	330-540	275-420
1 to3 years	450-700	250-500
4 to 10 years	700-1000	440-800
11 to 17 years	900-1300	640-1250

Source: Reference 53.

One of the difficulties in restricting dietary P is due to the consumption of foods containing P additives, since they promote an increase of up to two times more P than unprocessed foods. When P intake needs to be tightly controlled, it is important carefully evaluating the amount of protein offered daily to avoid a hypoproteic diet that could lead to malnutrition. A normoprotein diet is recommended for children aiming for a normal growth rate, even though 50% of the protein sources are of high biological value^5^. 

The low-P diet should be individualized, so that it is not too restrictive and provides nutrients according to each patient's needs, considering food access, eating habits and preferences. These factors contribute to a better adherence to the dietary program, which should be guided by an experienced nutritionist. 

Frequently, the patient with CKD and hyperphosphatemia, despite adherence to diet, fails to achieve good control of P, being necessary to use P binders; in those undergoing hemodialysis, it is advisable to optimize hemodialysis, including the time and frequency of sessions. 

Calcium carbonate (CaCO3) is an effective, inexpensive P binder with few side effects, being the first choice for treatment; however, its use depends on serum Ca levels and the amount needed to control P levels, always careful to avoid hypercalcemia^54^. In addition, the solubility of CaCO3 is higher in acidic media, so it should not be offered together with sodium bicarbonate. Some centers prefer calcium acetate considering the solubility over a wider pH range and a slightly better efficacy than CaCO3 as a phosphorus binder, but this preparation presents greater side effects^55^. 

Sevelamer is a Ca-free P binder, which is equally effective as calcium binders, but may have gastrointestinal side effects. The recommended dose of sevelamer should be proportional to the P content of the diet, with 140 ± 86 mg/kg/day (5.38 ± 3.24 g/day) being suggested in the age group above 10 months and under 2 years old^56^. For children older than 2 years old and adolescents, the starting dose is 400 or 800 mg, 3 times a day, at the main meals, with the final mean of 140-163 mg/kg/day (5.38 to 6.7g/day)^57^ ^,^ ^58^. 

Regarding Ca, as previously mentioned in the text, bone balance changes throughout life, depending on the relative rates of bone formation and resorption. Management of oral and/or enteral calcium intake in children with CKD is a challenging problem for physicians and nutritionists. Whereas an insufficient calcium supply may cause altered bone mineralization, increase the risk of fractures, and compromise growth, calcium overload may be associated with the risk of vascular calcification and cardiovascular events^5^. In the pediatric population, it is important to maintain Ca balance in order to ensure adequate growth and bone mass gain^59^. For patients with hypercalcemia, the use of Ca-based binders and vitamin D analogues should be discontinued until serum Ca levels are normalized^53^. 

Hypovitaminosis D is very frequent in patients with CKD, both in adults and children, and it could hardly be corrected by diet alone, since the intake of high vitamin D foods - such as cod liver oil, fish (tuna, salmon and sardines), liver, egg yolk, milk and fortified cheeses^53^ - is insufficient, for these foods are not commonly consumed by our population. Thus, we advise its supplementation when 25(OH)vitamin D levels are below 30 ng/mL^13^. Table 5 shows the treatment scheme for hypovitaminosis D^26^.

**Table 5.** Recommended doses of vitamin D2 or D3 supplementation in CKD stages 2-5D

Serum 25(OH)vitamin D level	Degree of deficiency	Dose of 25(OH)vitamin D (oral)	Duration	Serum control of 25(OH)vitamin D*
< 5 ng/mL	Severe	8,000 IU/day for 4 wks or 50,000 IU/week for 4 wks and after, 4,000 IU/day for 2 months or 50,000 IU, 2x/month for 2 months	3 months	CKD 2 to 5: 3 months CKD 5D: monthly
5-15 ng/mL	Moderate	4,000 IU/day for 3 months or 50,000 IU 2x/month for 3 months	3 months	CKD 2 to 5: 3 months CKD 5D: monthly
16-30 ng/mL	Insufficiency	2,000 IU/day or 50,000 IU/month	3 months	CKD 2 to 5: 3 months CKD 5D: monthly

Source: Reference 26.

## 2.2. Control of secondary hyperparathyroidism (SHPT)

2.2.1 For patients in CKD G3-5, the use of calcitriol and vitamin D is recommended for maintaining serum PTH levels in the appropriate range for the stage of CKD (Opinion).

2.2.2 For patients with CKD G3-5D and SHPT, we suggest the use of calcitriol or vitamin D analogues if serum Ca and P levels are within normal ranges (Evidence). 

2.2.3 For patients with CKD G5D and SHPT, whose serum Ca and/or P levels do not allow the use of calcitriol or vitamin D analogues, it is recommended initiating treatment with cinacalcet (Opinion).

2.2.4 Cinacalcet should not be used in patients with serum calcium below the reference value (Opinion).

2.2.5 For patients with CKD G5D and severe SHPT, the association of calcitriol or vitamin D analogues with cinacalcet is recommended for treatment optimization (Opinion).

2.2.6 For patients with CKD G5D and severe SHPT who do not respond to clinical treatment, parathyroidectomy is recommended (Evidence).

## Rational

PTH is an important biochemical marker of CKD-MBD, but although it is associated with changes in bone turnover and mineralization, the optimal serum levels are still a matter of debate. It is important to consider the linear growth potential of children, remembering that growth deficit is multifactorial and that PTH alone is not an optimal marker, with the association between PTH and growth varying^14^ ^,^ ^60^. This discussion is important for therapeutic decision-making, since, in any case, the treatment of SHPT relies on the control of phosphorus, calcium, and the use of vitamin D and its analogues, as discussed above.

In patients with advanced CKD, vitamin D analogues, such as calcitriol, are routinely used in the management of SHPT. Although some pediatric nephrologists use calcitriol as pulse therapy, this is not established in literature. Schmitt et al. have addressed the use of daily and intermittent calcitriol at comparable doses for one year, and concluded that there was no difference between groups, with both reducing PTH levels^61^ ^,^ ^62^. The initial scheme for the use of calcitriol is shown in Tables 6 and 7 ^26^.

**Table 6.** Recommendation for starting dose of calcitriol in pediatric patients with CKD G2-4

Serum Ca	Serum P	Alkaline phosphatase	Parathyroid hormone	Calcitriol
Normal	Normal	Normal/ Increased	< 2x the reference value	---
Normal	Normal	Normal/ Increased	> 2x the reference value	Consider the use (maximum 0.25 mcg/day)

Source: Adapted from reference 26.

**Table 7.** Recommendation for starting dose of calcitriol in pediatric patients with CKD 5-5D

Serum Ca	Serum P	Alkaline phosphatase	Parathyroid hormone	Calcitriol
Normal	Normal	Normal/ Increased	2 to 9x the reference value	Start with 0.25 mcg/day and progressively increase the dose up to 1 mcg/day
Normal	Normal	Normal/ Increased	≥ 9x the reference value	

Source: Adapted from reference 26.

Paricalcitol is a selective vitamin D analog, which differs from calcitriol by reducing side effects such as hypercalcemia and hyperphosphatemia. Pediatric studies have shown that the use of paricalcitol was effective in reducing PTH levels, without increasing Ca and P^63^ ^,^ ^64^. However, in Brazil, we only have the injectable formulation and it is not allowed for people under 18.

Cinacalcet hydrochloride is a modulator of Ca-sensing receptors (CaSR) that helps reduce PTH^65^ ^,^ ^66^, and it is available for pediatric use. In 2017, the European Medicines Agency approved the use of cinacalcet for the treatment of SHPT in dialysis children older than 3 years old who did not show adequate control of PTH levels with vitamin D analogues^67^. They recommend an initial dose of cinacalcet of ± 0.2 mg/kg/day based on dry weight, orally or via nasogastric tube, and it may be increased by 0.2 mg/kg/day up to a maximum daily dose of 2.5 mg/kg (not to exceed 180 mg). These increases depend on PTH and albumin-adjusted calcium levels, which should be > 2.2 mmol/L, requiring discontinuation of the drug if calcium levels are below this value. Dose titration intervals should be at least 4 weeks. The dose of cinacalcet should be reduced when PTH levels are between 100 and 150 pg/mL, or when they decrease too rapidly. Discontinuing cinacalcet when PTH concentrations are below the target range. Serum calcium levels should be monitored one week after initiation of therapy, weekly during tapering regimen, and at least monthly when the maintenance dose is established, in the stable patient. Serum PTH levels should be monitored monthly. It is important to alert caregivers to symptoms of hypocalcemia in children, such as paresthesia, myalgia, cramps, tetany, and seizures. In addition, provide guidance on the interaction with other drugs, and on the monitoring of serum calcium. In case the drug is discontinued, it might be restarted with a lower dose when serum calcium levels return to the upper limit of the normal range. Sohn et al. have assessed the use of cinacalcet, single dose, in children under 6 years old, demonstrating the safety of the medication^65^. Another study, also evaluating the safety of cinacalcet, has shown similar results with few adverse effects, such as hypocalcemia.

Children with severe hyperparathyroidism who do not respond to clinical treatment should be referred for parathyroidectomy (PTx). Subtotal parathyroidectomy is the recommended technique, since it decreases the risk of hypoparathyroidism and the complications after possible kidney transplantation. However, few centers in the country perform this procedure on children. 

Adequate monitoring of these children undergoing treatment for SHPT is important, as both active vitamin D and the use of calcimimetics may result in suppression of bone turnover, causing adynamic bone disease and contributing to growth deficits. In addition, if calcitriol may cause hypercalcemia, increasing the risk of vascular calcification, calcimimetics may lead to hypocalcemia, resulting in altered bone mineralization and arrhythmia^66^. 

We emphasize that PTH is merely one piece of the puzzle and that the growth of these children, bone comorbidities, as well as cardiovascular comorbidities, metabolic acidosis and anemia should be closely monitored for better quality of life and to reduce mortality rates.

## 2.3 Treatment with growth hormone

2.3.1 For infants with CKD G2-5D, it is recommended performing a linear growth assessment every 3 months (Evidence).

2.3.2 For children and adolescents with CKD G2-5D, it is recommended performing a linear growth assessment at least annually (Evidence).

2.3.3 For children and adolescents with CKD G2-5D, who progress with height deficits, treatment with human growth hormone is recommended, after nutritional assessment and correction of acidosis and CKD-MBD biochemical abnormalities (Evidence).

## Rational

Short stature has a negative impact on quality of life, self-esteem and social relationships and it is associated with increased mortality^68^ ^,^ ^69^. Stunting may be defined as height below the 3rd percentile for age and sex and growth velocity below the 25th percentile^70^. Around 40% of children with CKD have growth deficit and height below the 3rd percentile^71^. The etiology of growth deficit in CKD is multifactorial and includes intrauterine growth restriction, malnutrition, inflammation, mineral and bone disorder (MBD), metabolic acidosis, anemia, hormonal disturbances, among others^70^ ^,^ ^72^.

Randomized clinical trials have shown that GH stimulates growth in prepubertal children under conservative treatment, on dialysis, and after kidney transplantation ^73^. 

The European Society for Paediatric Nephrology recommends that children over 6 months with CKD stage 3-5D who have persistent stunting may be treated with GH, provided that other potentially treatable risk factors for growth retardation have been removed or treated, and that the child has growth potential and gets monitored. Contraindications to treatment are known hypersensitivity to the active substance or to any of the excipients, presence of PTH > 500 pg/mL, severe nonproliferative or proliferative diabetic retinopathy, during the first year after kidney transplantation, critically ill patients, and those with active malignant neoplasms. The dose of GH used in the observational studies was 28 to 30 international units (IU)/m^2^ per week (equivalent to 0.045 to 0.05 mg/kg per day)^72^.

A German study involving prepubertal CKD patients under conservative treatment and on dialysis has observed stature gain in response to treatment with GH and a positive association with residual kidney function and target height, but a negative association with age at treatment onset^74^. It is recommended to consider the cost-benefit and to inform the patient and guardians that the response to treatment is individual^72^. 

## 2.4 Assessment and treatment of bone disease in kidney transplantation (KTx)

2.4.1 Monitoring of Ca, P, AP, PTH and 25(OH)vitamin D after KTx

2.4.1.1 In the early period (0-3 months):

- Assess serum Ca and P weekly until stabilization (Evidence).
- Assess PTH and AP at the time of KTx (Opinion).
- Assess vitamin D (Evidence).

2.4.1.2 In the 3-12 month period, the frequency of assessment will depend on the magnitude of biochemical changes and the established therapeutics:

- Assess Ca and P monthly (Opinion).
- Assess PTH and AP in the 6th and 12th month (Opinion).
- Assess vitamin D every 6 months, or every 3 months in case of supplementation (Opinion).

2.1.1.3 In the late period (> 12 months), the frequency of assessment will depend on the renal graft function and on the stabilization of previously detected biochemical changes (Opinion).

- The same recommendations for CKD patients under conservative treatment should be followed in case of progressive loss of graft function (Evidence).
- CKD 1-3T: Ca, P, AP (6-12 months) and PTH annually.
- CKD 4T: Ca, P, AP, PTH (3-6 months).
- CKD 5T: Ca, P, AP (1-3 months) and PTH every 3 months.
- Monitor vitamin D every 6 months, or every 3 months in case of supplementation (Opinion).
- For patients with vascular and/or valve calcification, it is recommended undergoing annual echocardiograms (Opinion).

2.4.2 Treatment of CKD-MBD

- Hypovitaminosis D should be corrected using the same recommendations as for the population with risk factors (Evidence).
- Vitamin D supplementation should be discontinued in the presence of hypercalcemia (Evidence).
- For patients with persistent SHPT, consider the use of calcitriol.
- For patients with persistent SHPT and hypercalcemia, consider using cinalcalcet (Opinion).
- It is recommended considering bone biopsy for patients with SHPT who do not respond to usual treatment (Opinion).
- For patients with persistent SHPT who do not show adequate control with clinical treatment, parathyroidectomy is indicated (Evidence).

## Rational

Successful kidney transplantation corrects many of the abnormalities associated with CKD, but sometimes mineral and bone disorders remain and should be controlled. Essentially, post-transplant bone disease is due to previous bone disease acquired during the course of CKD, to bone impairment resulting from the use of immunosuppressants, especially corticosteroids^75^ and to graft survival time.

Hypophosphatemia occurs in most patients soon after transplantation, due to the phosphaturic action of FGF23 and PTH, but once kidney function stabilizes, P returns to normal levels^76^ ^,^ ^77^. Usually, spontaneous resolution of hypophosphatemia is expected, except in severe cases requiring replacement.

Hypercalcemia may occur in the first months after KTx, due to the persistence of SHPT, developed during the dialysis period. Although SHPT usually resolves within the first 12 months after KTx^78^ ^,^ ^79^, cinacalcet should be considered in those patients whose hypercalcemia persists during the 1st year of KTx^77^.

Hypovitaminosis D affects about 50% of transplant patients and should be corrected, following the same recommendations for CKD children before transplantation^80^.

The incidence of persistent SHPT (pSHPT) may vary around 50%; however, it is not frequent in children, since the functioning graft normalizes most of the metabolic changes. Thus, after KTx there is a progressive decrease in PTH levels, which often normalize after 6 months^78^ ^,^ ^81^. However, if the SHPT is persistent, the use of active vitamin D (calcitriol) is recommended. While on treatment with calcitriol or cinacalcet, some caution should be taken to avoid causing adynamic bone disease^82^ ^,^ ^83^, remembering that some patients have low bone turnover even with moderately elevated serum PTH levels^84^.

Bone biopsy data from kidney transplanted children with stable graft function indicate that 67% of the patients have normal bone formation, 10% have adynamic bone disease, and 23% pSHPT^8^ ^5^. 

PTx should be considered when patients have pSHPT with serum Ca levels persistently above 12.5 mg/dL for more than 12 months post KTx^86^ and no response to cinacalcet.

Osteonecrosis is the most debilitating skeletal complication related to organ transplantation. It affects about 15% of patients in the first 3 years after transplantation. Osteonecrosis, which also occurs after transplantation of other organs, infers that glucocorticoids play a critical role in the pathogenesis of this disorder^80^.

 A significant bone loss may occur early, about 3 to 6 months after KTx, and several factors are involved, such as persistent SHPT, prolonged immobilization, kidney function, and mainly the use of immunosuppressants, especially corticosteroids^87^. Another consideration is the daily and cumulative dose of glucocorticoids, which seems to be inversely related to the post-transplant growth rate. The administration of corticosteroids every other day improves growth in children, and the growth is even more pronounced when steroids are completely stopped^88^. 

In adults, DXA is able to predict fractures, and the current guideline recommends performing it in transplant patients as well. For pediatric transplant patients, normal values of bone mineral density might be obtained by DXA, provided that they are corrected for the degree of growth retardation. However, DXA does not determine the risk of fracture, and in clinical practice, the use of this test in KTx patients is also not recommended by the Pediatric CKD-MBD Guidelines.

The incidence of fracture in the first 6 months post-KTx is around 10%. Despite improvement in SHPT, normalization of muscle mass and trabecular bone within 12 months, the action of corticosteroids promotes persistent deficits in cortical bone dimensions and in bone strength^89^.

Cardiovascular disease remains the leading cause of death post-KTx. In the post-transplant period, the presence of hypertension is strongly associated to increased intima-media thickness and low vessel distensibility in children assessed by ultrasound^90^. Alterations in mineral metabolism also contribute to cardiovascular disease, considering that some degree of impaired renal function persists in most patients, even with a functioning graft. Thus, the assessment of these changes within the first 12 months post-KTx, and whenever there is loss of renal function, is fundamental.

 The KTx does not reverse vascular calcification, nevertheless it might be avoided when it is possible to perform preemptive KTx. We know that the optimal scenario for successful KTx would be the control of bone disease in the pre-transplant period.

In conclusion, mineral and bone disorder is frequent, serious, and difficult to treat. Although evidence is scarce, we have endeavored to review the most current recommendations, and hope that this guideline may contribute as a guide for the assessment and treatment of children and adolescents with CKD-MBD.
